# Cost-effectiveness analysis of rapid diagnostic tests for G6PD deficiency in patients with *Plasmodium vivax* malaria in the Brazilian Amazon

**DOI:** 10.1186/s12936-016-1140-x

**Published:** 2016-02-11

**Authors:** Henry M. Peixoto, Marcelo A. M. Brito, Gustavo A. S. Romero, Wuelton M. Monteiro, Marcus V. G. de Lacerda, Maria R. F. de Oliveira

**Affiliations:** Center for Tropical Medicine, University of Brasília, Brasília, Federal District Brazil; University Centre of Brasília, Brasília, Federal District Brazil; National Institute for Science and Technology for Health Technology Assessment (IATS/CNPq), Porto Alegre, Rio Grande do Sul Brazil; Tropical Medicine Foundation Dr. Heitor Vieira Dourado, Manaus, Amazonas Brazil; University of the State of Amazonas, Manaus, Amazonas Brazil; Instituto Leônidas e Maria Deane, FIOCRUZ, Manaus, Amazonas Brazil

**Keywords:** Cost-effectiveness analysis, Glucose-6-phosphate dehydrogenase deficiency, Primaquine, Haemolysis, Malaria, *Plasmodium vivax*, Economic analysis

## Abstract

**Background:**

The use of primaquine (PQ) for radical treatment of *Plasmodium vivax* in carriers of G6PD deficiency (G6PDd) constitutes the main factor associated with severe haemolysis in G6PDd. The current study aimed to estimate the incremental cost-effectiveness ratio of using a rapid diagnostic test (RDT) to detect G6PDd in male patients with *P.vivax* malaria in the Brazilian Amazon, in comparison with the routine indicated by the Programme for Malaria Control, which does not include this evaluation.

**Methods:**

A cost-effectiveness analysis of estimated RDT use was carried out for the Brazilian Amazon for the year 2013, considering the perspective of the Brazilian Public Health System. Using decision trees, estimates were compared for two different RDT strategies for G6PDd in male individuals infected with *P. vivax* before being prescribed PQ, with the routine indicated in Brazil, which does not include prior diagnosis of G6PDd. The first strategy considered the combined use of RDT BinaxNOW^®^ G6PD (BX-G6PD) in municipalities with more than 100,000 inhabitants and the routine programme (RP) for the other municipalities. Operational limitations related to the required temperature control and venous blood collection currently restrict the use of RDT BX-G6PD in small municipalities. The second strategy considered the use of the RDT CareStart™ G6PD (CS-G6PD) in 100 % of the municipalities. The analysis was carried out for the outcomes: “adequately diagnosed case” and “hospitalization avoided”.

**Results:**

For the outcome “adequately diagnosed case”, comparing the RDT strategies based on RDT with the routine control programme (RP), the CS-G6PD strategy was the most cost-effective, with BX-G6PD extendedly dominating (the ICER of BX-G6PD compared with RP was higher than the ICER of CS-G6PD compared with RP). CS-G6PD dominated the other strategies for the “hospitalization avoided” outcome.

**Conclusion:**

The CS-G6PD strategy is cost-effective for adequately diagnosing cases and avoiding hospitalization. This information can help in decision-making, both in incorporating prior diagnosis in the use of PQ and to promote greater safety among G6PD deficient individuals in the Brazilian Amazon *P. vivax* endemic areas.

## Background

The use of primaquine (PQ) for radical treatment of *Plasmodium vivax* in carriers of G6PD deficiency (G6PDd) constitutes the main factor associated with severe haemolysis, hospitalization and death in G6PDd individuals in Latin America, especially in males; cases of severe adverse events in females induced by PQ, have not been reported in the Brazilian Amazon [1, 2]. In Brazil, PQ is administered to prevent relapses of *P. vivax* malaria and to reduce its transmission [3]. However, this radical cure treatment of *P. vivax* malaria with PQ represents a risk for G6PDd carriers, since the Brazilian malaria national control programme does not recommend a routine G6PDd diagnosis before treatment starts. More than 99 % of the 178,600 new Brazilian cases of malaria in 2013 were reported in the Amazon, of which 81 % were caused by *P. vivax* [4, 5]. The estimates are approximately 6000 hospitalizations per year for individuals with G6PDd as a result of using PQ, creating a substantial burden, of an estimated costing of US$ 4,858,108.87 per year, for the Brazilian Public Health Service (Sistema Único de Saúde/SUS) [6]. In this context, detecting G6PDd before PQ is prescribed could contribute to improving treatment safety in people with G6PDd, preventing harm done to the efforts to control and eliminate malaria. In Brazilian Amazon, the prevalence of G6PDd in males was estimated at 4.5 % [7]. The identification and implementation of a rapid, efficient and feasible diagnostic method in field conditions is a priority **[**8–11].

The gold standard G6PDd diagnostic test is a quantitative spectrophotometric assay that requires laboratory setting, complex equipment and specialized staff. In less sophisticated settings, the fluorescent spot test has been widely recommended because it is easy to execute. However, this test also requires specialized equipment, such as an ultraviolet bulb, a water bath and a micropipette. Thus, neither of these two tests can be considered appropriate for field conditions in most of the malarial areas in the Amazon region [8, 12–14], and they are not used in routine services.

Two RDTs have been evaluated to detect G6PDd in field conditions under different situations: BinaxNOW^®^ G6PD (BX-G6PD) and CareStart™ G6PD (CS-G6PD). Both are qualitative chromatographic tests that are easily read visually, require minimal training and results can be obtained within 10 min [12, 15, 16].

BX-G6PD presents operational limitations related to temperature, as it provides reliable results only when performed at 18–25 °C range, and the sample required is venous blood [15, 17]. A recently reported multicentre study that included the city of Manaus found a sensitivity of 54.6 % and a specificity of 100 % for this test in malaria patients using a cut-off value corresponding to 60 % of normal activity [16]. The authors mentioned unpublished data for a cut-off of 40 % of normal activity the sensitivity and specificity were, respectively, 66.6 and 100 %.

CS-G6PD requires capillary blood samples collected from sterile finger pricks and results are not affected by high temperatures during its conservation and execution [12]. When this test was used for patients with malaria in Manaus, sensitivity was 45.45 % and specificity 98.71 %, for a cut-off value of 40 % of normal enzyme activity (Brito et al, Personal communication, 2015). In the general population of villages in Cambodia, this cut-off value the test showed a sensitivity and specificity of 93.3 and 97.4 % respectively [18].

The absence of cost-effectiveness analysis comparing the use of RDTs for G6PDd diagnosis with the *status quo*, where testing before PQ prescription is not performed, is an important gap that makes it difficult to recommend these tests [13, 14, 19]. The absence of a G6PDd diagnosis also represents an obstacle for the future use of tafenoquine, a new promising 8-aminoquinoline that has shown high efficacy in radical cure of *P*. *vivax* malaria when administered as a single dose [6, 19, 20]. Therefore, the present cost-effectiveness study was carried out with the objective of estimating the incremental cost-effectiveness ratio (ICER) of introducing a RDT to detect G6PDd in male patients carrying *P. vivax* in the Brazilian Amazon, in comparison with the routine programme (RP) of the Brazil’s Malaria Control Programme.

## Methods

A cost-effectiveness analysis (CEA) was done from the perspective of the Brazilian Public Health Service (SUS) for 2013. The study area covers the Brazilian Amazon, composed of 775 municipalities distributed in nine States (Acre, Amapá, Amazonas, Maranhão, Mato Grosso, Tocantins, Rondônia, Roraima and Pará), where the population is approximately 21 million, of whom 10 million live in 34 municipalities with more than 100,000 inhabitants [21]. In the region, 427 municipalities registered cases of *P. vivax* malaria in 2013 [4].

Two G6PDd RDT strategies were compared, in male individuals infected with *P. vivax* before they were prescribed PQ, with the RP stipulated in Brazil, without prior diagnosis of G6PDd. Females were excluded since in Latin America clinically important manifestations occur mainly in males (cases of severe adverse events induced by PQ, have not been reported in the study region). The first strategy was based on the combined use of BX-G6PD in the municipalities with more than 100,000 inhabitants and the RP in other municipalities (BX-G6PD strategy). Operational limitations related to the required temperature control and venous blood collection restrict the use of RDT BX-G6PD in small municipalities. The second was based on CS-G6PD (strategy CS-G6PD) considering its use in 100 % of the municipalities. The analysis was carried out for the outcomes: “adequately diagnosed case” and “hospitalization avoided”.

### Assumptions

This analysis used the assumptions previously detailed by Peixoto et al. [6], related to the diagnosis of malaria, the treatment of *P. vivax* and assistance to G6PDd carriers with severe adverse events associated with PQ, except the cost of hospital staff. In the current study the total mean cost of the Authorization for Hospitalization (AIH) includes the payment of professional and hospital services done by SUS. It was also assumed that the diagnostic units were able to store and carry out tests at the temperature recommended by the manufacturers, and that the execution of CS-G6PD would be carried out by health agents and that of BX-G6PD by nursing technicians.

### The decision model

**Fig. 1:**
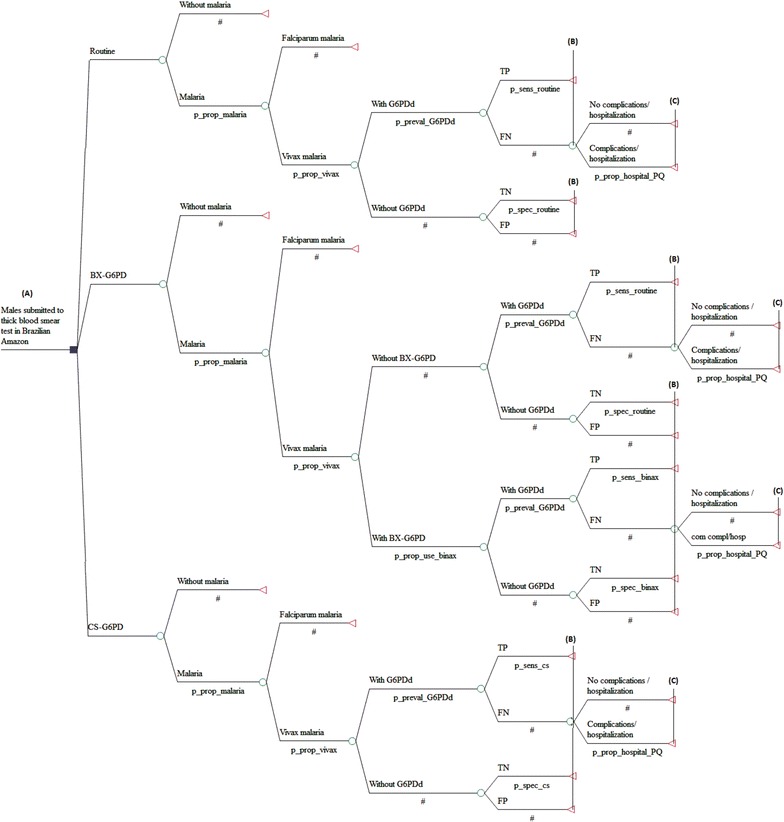
Decision tree for the outcomes: adequately diagnosed case (*A*–*B*) and avoided hospitalization (*A*–*C*). Brazilian Amazon, 2013

Decision trees were used to compare the strategies being evaluated (Fig. 1). A hypothetical cohort was simulated from all male patients who presented fever and requested diagnosis for malaria, considering its various probability nodes. After the malaria thick blood smear test, the patient was submitted to the probability of having or not having malaria, followed by the type of malaria—*P*. *vivax* or *P*. *falciparum*—and then to the probability of carrying out a diagnosis with one of the RDTs for G6PDd or to the RP without G6PDd diagnosis. The individual infected with *P. vivax* might or might not carry the deficiency, and this estimate was based on the prevalence of G6PDd in the Amazon Region.

After carrying out one of the tests for G6PDd, the probabilities were taken relative to the sensitivity and specificity of the diagnostic strategy used, with true positives (TP), false positives (FP), true negatives (TN) and false negatives (FN) results. For RP, the model assumes that the strategy is not able to diagnose the G6PDd, since it does not uses diagnostic test for this purpose. After use of the diagnostic test, TP and TN results were considered adequately diagnosed cases, whereas FP and FN were considered incorrectly diagnosed cases. After diagnosis, the hypothetical cohort was analysed by the probability of hospitalization. It was assumed that all individuals classified as FN and TN were submitted to treatment with PQ, and those identified as TP and FP were not. Thus, the carrier of G6PDd who uses PQ (FN) is submitted to the probability of not being hospitalized (hospitalization avoided) or hospitalization due to severe adverse events provoked by the medication. All costs and epidemiological parameters used in the decision model are shown in Tables 1 and 2.

**Table 1:** Cost components and unit costs considered in the analytical model

Items	Cost per patient (unit costs, US$)		Sources base-case/variations
	Base-case	Variations	
Diagnosis of *P*. *vivax* malária			
Thick blood smear^a^	1.31	0.27–2.00	[40] / [35]–[40]
Microscope^a^	0.75	0.60–0.90	[41] /± 20 %
Microscope maintenance^a^	0.06	0.05–0.07	[41] /± 20 %
Health workers^a^	3.48	2.78–4.18	[42] /± 20 %
Annual training (microscopist)^a^	5.67	4.54–6.80	[35] /± 20 %
Drug treatment for *P. vivax* (therapeutic scheme)			
Males without G6PDd^b^	0.23	0.18–0.28	[43] /± 20 %
Males with G6PDd^b^	0.12	0.10–0.14	[43] /± 20 %
Males with G6PDd/prophylactic treatment^b^	0.28	0.22–0.34	[43] /± 20 %
G6PDd diagnosis			
Training (RDT)^c^	0.80	0.64–0.96	[35] /± 20 %
CS-G6PD			
Health worker^d^	1.74	1.39–2.09	[42] /± 20 %
One test	1.50	1.20–1.80	[44, 45] /± 20 %
Supplies^e^	0.10	0.08–0.12	[46] / [46]–[46]
BX-G6PD			
Health worker^d^	2.04	1.63–2.45	[42] /± 20 %
One test	5.88	4.70–7.06	[47] /± 20 %
Supplies^e^	1.09	0.87–1.31	[45, 46] / [46]–[46]
Assistance to carriers of G6PDd treated with PQ			
Pre-admission tests^a^	10.69	9.65–11.38	[4, 40] / [4, 40]
Medical appointments^a^	4.63	–	[48] /–
Hospitalization (AIH)^f^	198.81	159.05–238.57	[49] /± 20 %
Hospital food^a^	68.72	54.98–82.46	[50] /± 20 %

Brazilian Amazon, 2013

^a^Costing previously detailed by Peixoto et al. [6]

^b^Therapeutic schemes: chloroquine for 3 days and PQ for 7 days (males without G6PDd); chloroquine for 3 days (males with G6PDd) and prevention of relapse with chloroquine weekly for 12 weeks (prophylactic treatment)

^c^Based on the cost of one training period per year per municipality for the diagnosis of malaria with RDT OptiMal^®^ in the Brazilian Amazon

^d^Cost per test based on the average salary of Amazonas State in 2013

^e^Variation obtained from the minimum and maximum cost identified in the time series of prices of bank (BPS) within the Ministry of Health (MoH) in 2013

^f^Cost obtained through the analysis of 108 files from the Hospital Information System (SIH/SUS) of the Brazilian Amazon in 2013

**Table 2:** Epidemiological parameters considered in the analytic models. Brazilian Amazon, 2013

Parameter	Base-case	Variations	Sources base-case/variations
Proportion of malaria among febrile males seeking diagnosis^a^	0.0886	0.0881–0.0891	[4] /IC 95 %
Proportion of malaria cases due to *P. vivax* in male patients^a^	0.835	0.833–0.837	[4] /IC 95 %
Prevalence of G6PDd in male patients	0.045	0.025–0.056	[7] /f]–[7] IC 95 %
Proportion of males with G6PDd hospitalized after treatment with PQ^b^	0.943	0.744–1	[39] /± 20 %
Proportion of the use of BX-G6PD^c^	0.184	0.182–0.187	[4, 21] /IC 95 %
Sensitivity of CS-G6PD^d^	0.455	0.213–0.720	f/IC 95 %
Specificity of CS-G6PD^d^	0.987	0.967–0.995	f/IC 95 %
Sensitivity of BX-G6PD^d, e^	0.667	0.301–0.921	[16] /IC 95 %
Specificity of BX-G6PD^d, e^	1	0.989–1	[16] /IC 95 %

^a^Parameter obtained from the analysis of the databanks from the nine States of the Brazilian Amazon

^b^Parameter obtained from follow-up of patients with G6PDd in FMT-HVD

^c^Proportion corresponding to individuals diagnosed with *P*. *vivax* malaria, residents of municipalities with more than 100,000 inhabitants

^d^The cutoff point: 40 % of the median enzyme activity in the sample

^e^The base-case and the variation considering data from Brazil and Peru

^f^Brito et al. Personal Communication, 2015

### Costs

For the outcome “adequately diagnosed case” the health costs arising from malaria diagnosis among male individuals and the diagnosis of G6PDd in individuals with *P*. *vivax* malaria were considered. For the outcome “hospitalization avoided” medication-based treatment for *P*. *vivax* malaria and the costs of health assistance for those undergoing severe adverse events associated with the use of PQ in G6PDd carriers were added on (Table 1). The costs obtained in Brazilian currency (reais, R$) were converted to US dollars (US$) using the mean value of the official exchange rate for the year 2013 (R$ 2.16 per dollar) [22]. The costs identified in other years were adjusted based on the official inflation rates estimated by the accumulated National Consumer Price Index (IPCA) [23].

### Epidemiological parameters

Epidemiological data and those of services related to malaria and to G6PDd within the context of malaria were obtained from the Malaria Epidemiological Vigilance Information System (SIVEP/Malária) [4], from the scientific literature and from unpublished documents and databanks at the Tropical Medicine Foundation Dr. Heitor Vieira Dourado (FMT-HVD) (Table 2).

### Cost-effectiveness and sensitivity analysis

By means of the decision models, the final costs and the effectiveness of the strategies were obtained, as well as the incremental cost-effectiveness ratios (ICER) for “adequately diagnosed case” and “hospitalization avoided”, considering the parameters used in base-case. Univariate and multivariate sensitivity analyses were carried out for all the epidemiological and cost parameters, except for the cost of the specialized medical consultation, which is a fixed cost in the health system and did not undergo alterations during the study period.

Tornado diagrams were based on the individual impact of each of the variables on the ICER to demonstrate the results from univariate analysis. The probabilistic sensitivity analysis (PSA) was carried out by means of the second-order Monte Carlo simulation, which evaluated the simultaneous impact of all the mentioned variables with regard to ICER. The following distributions were attributed to the entry parameters: beta distribution for the probability parameters and gamma or triangular distribution for the cost parameters. Finally, considering the three strategies evaluated for each of the outcomes, 10,000 interactions were carried out to produce acceptability curves based on a willingness to pay of US$ 6480 per additional outcome, which is equivalent to the *per capita* GDP for the Amazon Region. The software TreeAge Pro^®^ 2013 (TreeAge Pro Inc, Williamstown, MA, USA) was used to construct the decision model, for the calculation of the ICERs and for sensitivity analyses.

## Ethical considerations

The research project was submitted to the Ethics Committee of the Faculty of Health Sciences at the University of Brasilia and approved on May 14th 2013 (case number 276,522).

## Results

The strategy based on the use of CS-G6PD in 100 % of the population was more cost-effective when compared to RP and the strategy that combines BX-G6PD and RP (BX-G6PD strategy). The BX-G6PD strategy was extendedly dominated (the ICER of BX-G6PD compared with RP was higher than the ICER of CS-G6PD compared with RP) and the strategy CS-G6PD was the most cost-effective for the outcome “adequately diagnosed case”. For “hospitalization avoided”, the RP and BX-G6PD strategies were dominated by the CS-G6PD strategy (Table 3).

**Table 3:** Results of the cost-effectiveness analysis of the strategies “RP”, “CS-G6PD” and “BX-G6PD”, according to the outcomes assessed in the Brazilian Amazon, 2013

Strategy	Effectiveness	Incremental effectiveness	Cost (US$)	Incremental cost (US$)	ICER (US$)
Outcome: adequately diagnosed case					
RP	0	–	11.270	–	–
BX-G6PD (vs. RP)	0.0135	0.0135	11.404	0.134	9.96
CS-G6PD (vs. RP)	0.0716	0.0716	11.578	0.308	4.30
CS-G6PD (vs. BX-G6PD)	0.0716	0.0581	11.578	0.174	2.99
Outcome: hospitalization avoided					
CS-G6PD	0.0723	–	12.078	–	Dominant
BX-G6PD (vs. CS-G6PD)	0.0712	−0.0010	12.200	0.122	Dominated
RP (vs. CS-G6PD)	0.0708	−0.0014	12.175	0.097	Dominated
BX-G6PD (vs. RP)	0.0712	0.0004	12.200	0.025	63.92

**Fig. 2:**
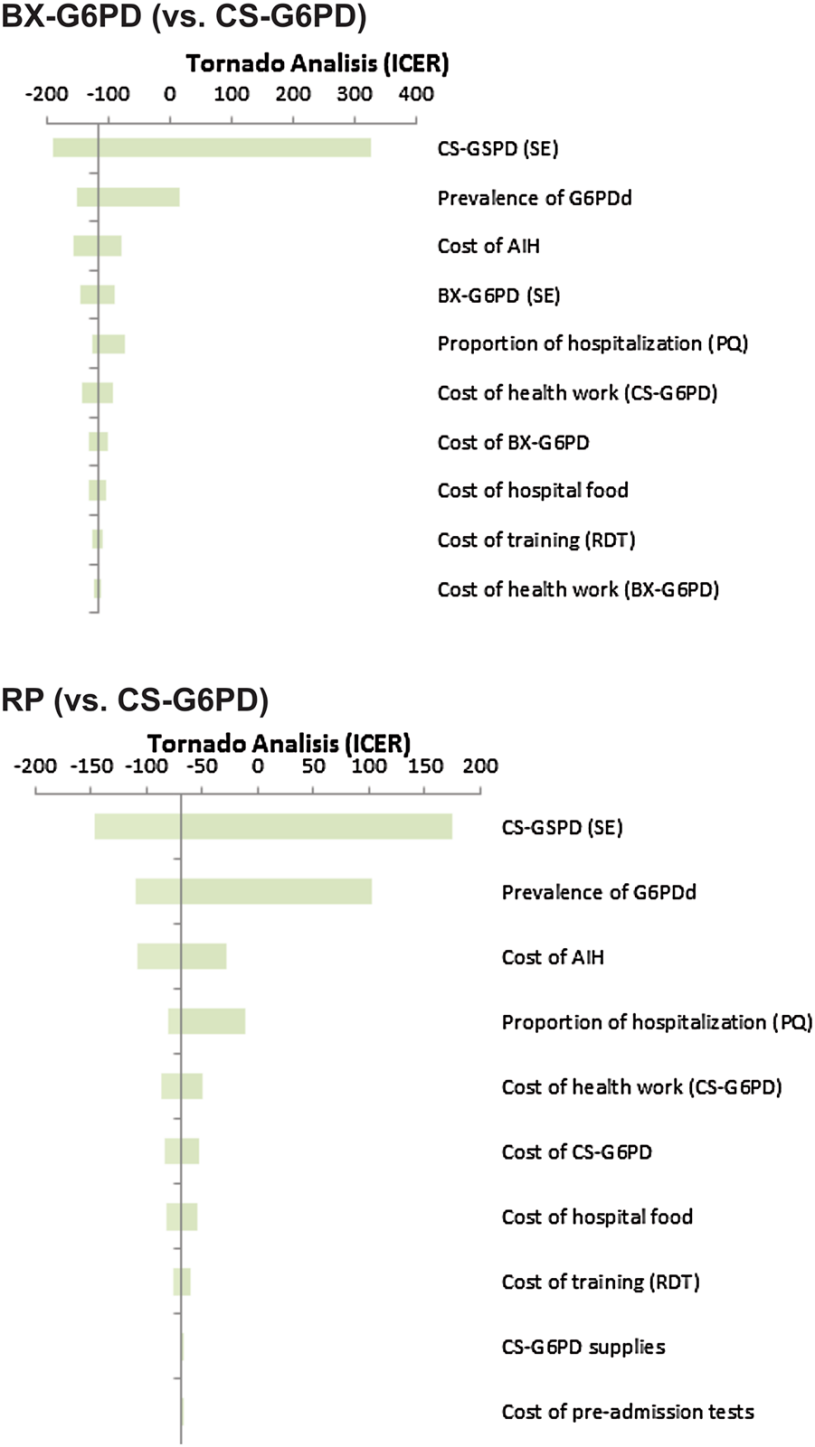
Univariate sensitivity analysis for the outcome hospitalization avoided, based on the individual impact of each of the variables on the ICER. Brazilian Amazon, 2013

The univariate sensitivity analyses for the outcome “adequately diagnosed case” showed that variations in the epidemiological and cost parameters did not have an important impact on the ICERs, whose greatest variation was only US$ 0.45. For “hospitalization avoided” the CS-G6PD strategy remained dominant in relation to the BX-G6PD and RP strategies, as evidenced by negative ICERs, supporting a greater effectiveness and lower cost for the CS-G6PD strategy. Exceptions occurred only in the sensitivity analysis of the variables sensitivity of CS-G6PD and prevalence of G6PDd, with positive ICERs (Fig. 2).

**Fig. 3:**
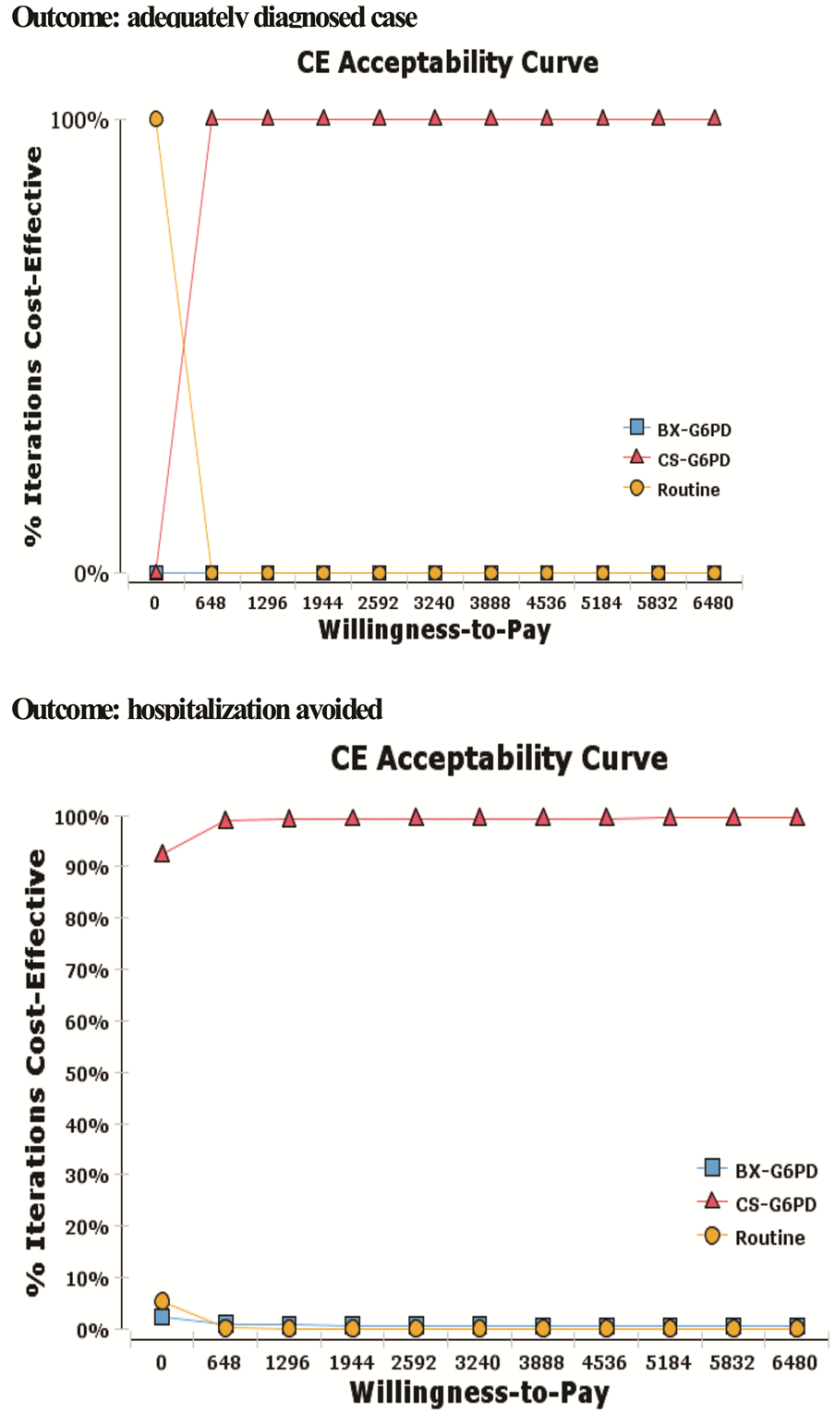
Acceptability curves of the three evaluated strategies in accordance with the outcome. Brazilian Amazon, 2013

Two acceptability curves were generated for the PSA (Fig. 3), for the outcomes “adequately diagnosed case” and “hospitalization avoided” respectively. For both curves, the strategy based on CS-G6PD presented a high probability of being cost-effective, at 10 % of the limit considered in the analysis.

Additionally, ICERs were estimated considering the highest (18.6 %) and the lowest (4.3 %) proportion of malaria in individuals submitted to the thick blood smear test among the States evaluated. The results demonstrate that the CS-G6PD strategy continues to be the most cost-effective one for appropriate diagnosis of G6PDd, presenting for both proportions the same ICER as that identified in the base-case when compared to the RP. For the outcome “hospitalization avoided”, the CS-G6PD strategy continued to dominate the others. The second analysis of additional sensitivity simulated the amplification of the use of BX-G6PD for the municipalities with more than 50,000 inhabitants (where 40.9 % of the cases of *P*. *vivax* malaria in men occurred). Again, the results did not cause significant modifications in the ICERs, and the CS-G6PD strategy continued to be the most cost-effective for the outcome “adequately diagnosed case” and was dominant for “hospitalization avoided”.

## Discussion

Although malaria continues to be one of the main public health problems in the world, there have been some successful results from control efforts in recent years, leading some endemic regions to consider the possibility of malaria eliminating [24]. Tatem et al. showed that the Americas present great potential for elimination of *Plasmodium falciparum,* as well as operational conditions for controlling *P. vivax*. In this context, for the regional efforts directed to eliminating malaria to be successful, the strategies directed to combating *P. vivax* have taken on an even greater importance [9, 25].

The elimination of *P*. *vivax* malaria will only be possible with the effective use of an 8-aminoquinoline for the radical treatment of latent *P. vivax* infections [13, 19]. However, as the use of this type of anti-malarials threatens the safety of G6PDd individuals, previous diagnosis of this condition in patients with *P*. *vivax* malaria needs discussion [13, 19], and it is vital that cost-effective diagnostic strategies are identified [1, 6, 11].

The RDTs considered in the study have different accuracies and integrate different strategies. The cost associated with performing each test (training and salary of health workers, test kits and supplies) was also different: US$ 9.81 for BX-G6PD and US$ 4.14 for CS-G6PD.

The present study constitutes the first CEA of strategies based on the two main RDTs for diagnosing G6PDd that are available and feasible under field conditions. These were compared to the routine strategy for prescribing PQ without prior diagnosis of the deficiency in the Brazilian Amazon. The results indicate that the strategy based on the use of CS-G6PD is cost-effective, for the outcome “adequately diagnosis of G6PDd”, whose ICERs were lower than 1 % of the *per capita* GDP of the region, and also for the outcome “hospitalization avoided”, for which it was dominant.

A hypothetical 2013 cohort of 1,188,523 males presenting with fever and requesting diagnosis for malaria would be administered 87,928 RDT CS-G6PD in males with *P. vivax* (RDT in 100 % of the municipalities) or 16,179 RDT BX-G6PD in males with *P. vivax*, based on combined use with RP (RDT in 18.4 % of the municipalities), which could prevent, respectively, 1808 hospitalizations (a cost of US$ 511,522) and 488 hospitalizations (US$ 137,974).

The analysis assumed the values of sensitivity and specificity obtained in studies carried out with patients diagnosed with malaria (Table 2). These estimate a reduction in the accuracy of the RDTs evaluated when compared to the accuracy estimated in studies with the general population. This may occur due to the greater number of new erythrocytes produced as a result of the acute haemolysis provoked by malaria, since new erythrocytes present more G6PD enzymatic activity when compared to mature cells, which may lead to false negative results [16, 26].

A recent study that evaluated the new generation of CS-G6PD demonstrated an improvement in its sensitivity for the general population in Cambodia [18], which could also occur in individuals with malaria, making the strategy even more cost-effective. Corroborating these promising results, another study in an area with high incidence of malaria in Africa recently estimated 100 % sensitivity in the general male population [27].

When the uncertainties concerning epidemiological and cost parameters were tested by means of the univariate analysis of sensitivity, the performance of the strategy based on CS-G6PD was not affected in an important way. The lowest variations in the sensitivity parameter of CS-G6PD and the prevalence parameter of G6PDd altered the dominance relationship of the CS-G6PD strategy for the outcome “hospitalization avoided”, but the strategy remained cost-effective.

The acceptability curves indicate that the probability of the strategy based on CS-G6PD being cost-effective is of approximately 100 %, for both outcomes, at 10 % of the limit of willingness to pay. For the second outcome, when the willingness to pay is US$ 0.00, the probability is over 90 %, demonstrating that the stochastic and simultaneous variation of the parameters for epidemiology and costs used indicates a high probability of the CS-G6PD strategy being cost-effective, despite the uncertainties inherent in the analytical model.

Decision-makers are being faced with increasingly difficult decisions when they consider the addition of new technologies to national malaria control program [28]. Aiming to help in decision-making, various economic studies have examined aspects related to primary prevention, diagnosis and treatment of malaria, with emphasis on vaccines [29, 30], mosquito nets impregnated with insecticides [31, 32], mass screening followed by treatment [33], use of RDTs to diagnose the infection [34, 35] and the use of schemes for chemo-prophylaxis and treatment [36–38]. Although there are no published studies on cost-effectiveness for G6PDd diagnosis in connection with malaria, recent publications have discussed the urgency of evaluating the efficiency of RDTs used to detect G6PDd so as to guarantee safety in the treatment of these patients [11, 13, 14].

This study presents some limitations, such as the fact that some epidemiological and cost parameters arise from one Amazon state arguing that results can be extrapolated to Brazilian Amazon. However, in the analysis of sensitivity, which includes plausible variations for the region studied, there were no important changes in the ICERs. The prevalence of G6PDd, the costs of hospital food and the salaries of the professionals involved in the RDT were generalized from data on the State of Amazonas, and the proportions of hospitalizations (94.29 %) were calculated from a cohort of G6PDd carriers who were monitored at FMT-HVD [39]. This represents the situation at a center of excellence, differing from the context in small towns of the Brazilian Amazon. Thus, the proportion of hospitalizations may have been overestimated, since in these places there is limited access to health services with moderate to high technological density [6].

Within this scope, the present study estimated a cost-effectiveness ratio that is favorable to the use of RDTs for G6PDd in the Brazilian Amazon, in particular CS-G6PD. Decision-makers in the National Programme of Prevention and Control of Malaria in the region should consider implementation of such RDT strategies for G6PDd diagnosis. The incorporation of RDTs will make the use of 8-aminoquinolines safer and may represent an increase in the effectiveness of *P*. *vivax* malaria control in Brazil.

## Conclusion

The strategy based on CS-G6PD is cost-effective in diagnosing G6PDd and avoiding hospitalization. These results may help in future decision-making related to the use of CS-G6PD in the Brazilian Amazon when considering a safer *P*. *vivax* malaria treatment in the region for G6PDd individuals.

## Abbreviations

G6PD: glucose-6-phosphate dehydrogenase
G6PDd: glucose-6-phosphate dehydrogenase deficiency
PQ: primaquine
RDT: rapid diagnostic test
RP: routine programme
CEA: cost-effectiveness analysis
SUS: Brazilian Public Health Service
IPCA: National Consumer Price Index
ICER: incremental cost-effectiveness ratios
PSA: probabilistic sensitivity analysis

## References

[1] Monteiro WM, Franca GP, Melo GC, Queiroz ALM, Brito M, Peixoto HM (2014). Clinical complications of G6PD deficiency in Latin American and Caribbean populations: systematic review and implications for malaria elimination programmes. Malar J.

[2] Lacerda MVG, Fragoso SCP, Alecrim MGC, Alexandre MAA, Magalhães BML, Siqueira AM (2012). Postmortem characterization of patients with clinical diagnosis of *Plasmodium vivax* malaria: to what extent does this parasite kill?. Clin Infect Dis.

[3] Ministério da Saúde: Guia prático de tratamento da malária no Brasil. Brasília; 2010.

[4] Ministério da Saúde: Sistema de informação de Vigilância Epidemiológica da Malária (SIVEP-Malária) http://portalweb04.saude.gov.br/sivep_malaria/default.asp.

[5] Ministério da Saúde: Sistema de Informação de Agravos de Notificação (SINAN) http://dtr2004.saude.gov.br/sinanweb/tabnet/dh?sinannet/malaria/bases/malabrnet.def.

[6] Peixoto HM, Brito MA, Romero GA, Monteiro WM, Lacerda MV, Oliveira MRF (2015). G6PD deficiency in male individuals infected by *Plasmodium vivax* malaria in the Brazilian Amazon: a cost study. Malar J.

[7] Santana MS, Monteiro WM, Siqueira AM, Costa MF, Sampaio V, Lacerda MV (2013). Glucose-6-phosphate dehydrogenase deficient variants are associated with reduced susceptibility to malaria in the Brazilian Amazon. Trans R Soc Trop Med Hyg.

[8] Kahn M, Larue N, Bansil P, Kalnoky M, McGray S, Domingo GJ (2013). Cryopreservation of glucose-6-phosphate dehydrogenase activity inside red blood cells: developing a specimen repository in support of development and evaluation of glucose-6-phosphate dehydrogenase deficiency tests. Malar J.

[9] Gething PW, Elyazar IRF, Moyes CL, Smith DL, Battle KE, Guerra CA (2012). A long neglected world malaria map: *Plasmodium vivax* endemicity in 2010. PLoS Negl Trop Dis.

[10] The malERA Consultative Group on Drugs (2011). A research agenda for malaria eradication: drugs. PLoS Med.

[11] Monteiro WM, Val FF, Siqueira AM, Franca GP, Sampaio VS, Melo GC (2014). G6PD deficiency in Latin America: systematic review on prevalence and variants. Mem Inst Oswaldo Cruz.

[12] Kim S, Nguon C, Guillard B, Duong S, Chy S, Sum S (2011). Performance of the carestart™ G6PD deficiency screening test, a point-of-care diagnostic for primaquine therapy screening. PLoS One.

[13] Domingo GJ, Satyagraha AW, Anvikar A, Baird K, Bancone G, Bansil P, Carter N (2013). G6PD testing in support of treatment and elimination of malaria: recommendations for evaluation of G6PD tests. Malar J.

[14] Von Seidlein L, Auburn S, Espino F, Shanks D, Cheng Q, McCarthy J (2013). Review of key knowledge gaps in glucose-6-phosphate dehydrogenase deficiency detection with regard to the safe clinical deployment of 8-aminoquinoline treatment regimens: a workshop report. Malar J.

[15] Tinley KE, Loughlin AM, Jepson A, Barnett ED (2010). Evaluation of a rapid qualitative enzyme chromatographic test for glucose-6-phosphate dehydrogenase deficiency. Am J Trop Med Hyg.

[16] Osorio L, Carter N, Arthur P, Bancone G, Gopalan S, Gupta SK (2015). Performance of BinaxNOW G6PD deficiency point-of-care diagnostic in P*. vivax*-infected subjects. Am J Trop Med Hyg.

[17] Eziefula AC, Gosling R, Hwang J, Hsiang MS, Bousema T, von Seidlein L (2012). Rationale for short course primaquine in Africa to interrupt malaria transmission. Malar J.

[18] Roca-Feltrer A, Khim N, Kim S, Chy S, Canier L, Kerleguer A (2014). Field trial evaluation of the performances of point-of-care tests for screening G6PD deficiency in Cambodia. PLoS One.

[19] Recht J, Ashley E, White N. Safety of 8-aminoquinoline antimalarial medicines. Geneva: World Health Organization; 2014. http://www.who.int/malaria/publications/atoz/9789241506977/en/.

[20] Llanos-Cuentas A, Lacerda MV, Rueangweerayut R, Krudsood S, Gupta SK, Kochar SK (2014). Tafenoquine plus chloroquine for the treatment and relapse prevention of Plasmodium vivax malaria (DETECTIVE): a multicentre, double-blind, randomised, phase 2b dose-selection study. Lancet.

[21] Superintendência do Desenvolvimento da Amazônia: Amazônia Legal http://www.sudam.gov.br/amazonia-legal/demografia/50-amazonialegal.

[22] Banco Central do Brasil: Taxas de Câmbio http://www4.bcb.gov.br/pec/taxas/port/ptaxnpesq.asp?id=txcotacao.

[23] Ministério do Planejamento Orçamento e Gestão: Sistema Nacional de Índices de Preços ao Consumidor http://www.ibge.gov.br/home/estatistica/indicadores/precos/inpc_ipca/defaultseriesHist.shtm.

[24] WHO (2014). From malaria control to malaria elimination: a manual for elimination scenario planning.

[25] Tatem AJ, Smith DL, Gething PW, Kabaria CW, Snow RW, Hay SI (2010). Ranking of elimination feasibility between malaria-endemic countries. Lancet.

[26] Cappellini MD, Fiorelli G (2008). Glucose-6-phosphate dehydrogenase deficiency. Lancet.

[27] Adu-Gyasi D, Asante KP, Newton S, Dosoo D, Amoako S, Adjei G (2015). Evaluation of the diagnostic accuracy of CareStart G6PD deficiency rapid diagnostic test (RDT) in a malaria endemic area in Ghana, Africa. PLoS One.

[28] Moorthy VS, Hutubessy R, Newman RD, Hombach J (2012). Decision-making on malaria vaccine introduction: the role of cost-effectiveness analyses. Bull World Health Organ.

[29] Seo MK, Baker P, Ngo KN-L (2014). Cost-effectiveness analysis of vaccinating children in Malawi with RTS, S vaccines in comparison with long-lasting insecticide-treated nets. Malar J.

[30] Tediosi F, Maire N, Penny M, Studer A, Smith TA (2009). Simulation of the cost-effectiveness of malaria vaccines. Malar J.

[31] Hanson K, Kikumbih N, Armstrong Schellenberg J, Mponda H, Nathan R, Lake S (2003). Cost-effectiveness of social marketing of insecticide-treated nets for malaria control in the United Republic of Tanzania. Bull World Health Organ.

[32] Paintain SL, Awini E, Addei S, Kukula V, Nikoi C, Sarpong D (2014). Evaluation of a universal long-lasting insecticidal net (LLIN) distribution campaign in Ghana: cost effectiveness of distribution and hang-up activities. Malar J.

[33] Crowell V, Briët OJ, Hardy D, Chitnis N, Maire N, Di Pasguale A (2012). Modeling the cost-effectiveness of mass screening and treatment for reducing *Plasmodium falciparum* malaria burden. Malar J.

[34] Oliveira MRF, Giozza SP, Peixoto HM, Romero GAS (2012). Cost-effectiveness of diagnostic for malaria in extra-Amazon Region. Brazil Malar J.

[35] Oliveira MRF, Gomes AC, Toscano CM (2010). Cost effectiveness of OptiMal^®^ rapid diagnostic test for malaria in remote areas of the Amazon Region. Brazil Malar J.

[36] Lubell Y, Riewpaiboon A, Dondorp AM, von Seidlein L, Mokuolu OA, Nansumba M (2011). Cost-effectiveness of parenteral artesunate for treating children with severe malaria in sub-Saharan Africa. Bull World Health Organ.

[37] Kyaw S, Drake T, Ruangveerayuth R, Chierakul W, White NJ, Newton PN (2014). Cost of treating inpatient falciparum malaria on the Thai-Myanmar border. Malar J.

[38] Massad E, Behrens BC, Coutinho FAB, Behrens RH (2011). Cost risk benefit analysis to support chemoprophylaxis policy for travellers to malaria endemic countries. Malar J.

[39] Fundação de Medicina Tropical Dr. Heitor Vieira Dourado (FMT-HVD). Banco de dados—deficiência da glicose-6-fosfato-desidrogenase (2009–2011). Manaus: FMT-HVD; 2011.

[40] Macauley C (2005). Aggressive active case detection: a malaria control strategy based on the Brazilian model. Soc Sci Med.

[41] Oliveira MRF. Cost-effectiveness analysis of rapid test for the diagnosis of new malaria cases in twelve endemic municipalities of the State of Pará. *PhD thesis*. Universidade de São Paulo, Faculdade de Saúde Pública; 2009.

[42] Secretaria de Estado da Saúde do Amazonas (SES/AM). Tabela de vencimentos e gratificação de saúde. Manaus: SES/AM; 2013.

[43] Secretaria de Vigilância em Saúde do Ministério da Saúde. Relação de compras de medicamentos para malária. Brasília, 2013.

[44] Bosman A, Cunningham J (2013). Proposal for an Evidence Review Group (ERG) on G6PD testing to support increased access to primaquine for radical cure of *Plasmodium vivax* and for malaria chemoprophylaxis.

[45] Fundação de Medicina Tropical Dr. Heitor Vieira Dourado (FMT-HVD): Relação de compras de insumos laboratoriais. Manaus; 2013.

[46] Ministério da Saúde: Banco de Preços em Saúde http://aplicacao.saude.gov.br/bps/login.jsf.

[47] Alere™: Informação fornecida pela chefia de produtos usados em doenças crônicas. São Paulo; 2014.

[48] Ministério da Saúde: Sistema de Gerenciamento da Tabela de Procedimentos, Medicamentos e OPM do SUS http://sigtap.datasus.gov.br/tabela-unificada/app/sec/inicio.jsp.

[49] Ministério da Saúde: Sistema de Informação Hospitalar Descentralizados do SUS http://www2.datasus.gov.br/SIHD/.

[50] Fundação de Medicina Tropical Dr (2013). Heitor Vieira Dourado (FMT-HVD): Custos com a nutrição hospitalar.

